# Meclofenamic Acid Restores Gefinitib Sensitivity by Downregulating Breast Cancer Resistance Protein and Multidrug Resistance Protein 7 *via* FTO/m6A-Demethylation/c-Myc in Non-Small Cell Lung Cancer

**DOI:** 10.3389/fonc.2022.870636

**Published:** 2022-04-21

**Authors:** Hui Chen, Bin Jia, Qiang Zhang, Yu Zhang

**Affiliations:** Department of Lung Cancer, Tianjin Medical University Cancer Institute and Hospital, National Clinical Research Center for Cancer, Key Laboratory of Cancer Prevention and Therapy, Tianjin’s Clinical Research Center for Cancer, Tianjin Lung Cancer Center, Tianjin, China

**Keywords:** meclofenamic acid, gefitinib, M^6^A, non-small cell lung cancer (NSCLC), drug resistance

## Abstract

**Background and Objective:**

Gefitinib (GE) is a first-line epidermal growth factor receptor (EGFR) tyrosine kinase inhibitor (TKI) for patients with advanced non-small cell lung cancer (NSCLC) carrying EGFR activating mutations. However, drug resistance limits the clinical efficacy of gefitinib and ultimately leads to extremely poor clinical benefit. Meclofenamic acid (MA) is a non-steroidal anti-inflammatory drug (NSAID) that relieves moderate and severe pain. In the present study, we aim to determine the MA sensibilization of GE in NSCLC.

**Methods:**

MTT assay was conducted to determine the synergistic effect of MA with GE in GE-sensitive and -resistant cell lines based on the Chou–Talalay method. The Annexin V-PI flow cytometry analysis was conducted to evaluate apoptosis. Western blot assay was used to detect alterations of EGFR downstream molecules. Tritium-labeled GE accumulation analysis was used to determine the efflux activity of GE. Dot blot assays were conducted to determine m6A levels after the MA and GE co-administration. Western blot evaluated the expression of FTO, c-Myc, MRP7, BCRP, and apoptotic proteins.

**Results:**

MA showed a significant synergistic effect with GE in GE-resistant NSCLC cells; co-administration of MA with GE induced caspase-related apoptosis in resistant NSCLC cells. Moreover, EGFR downstream molecules, including Akt and MAPKs pathways, were significantly inhibited by the MA-GE combination. Short-term incubation of MA did not alter the efflux of GE; however, after incubation for 24 h, the accumulation of tritium-labeled GE significantly increased. A mechanism study showed that co-administration of MA and GE significantly downregulated BCRP and MRP7 expression in GE-resistant cells; increased N6-methylation was also observed after co-administration. The FTO/c-Myc was determined as target pathways on MA and GE co-administration mechanisms.

**Conclusion:**

Our findings provide novel therapeutic approaches for GE-resistant NSCLC by combination use with MA through FTO-mediated N6-demethylation.

## Introduction

Though crucial progress for the management of non-small cell lung cancer (NSCLC) has been made in the last 20 years, the overall survival rates of NSCLC are still reasonably low. The 5-year overall survival of NSCLC is only 15% (1). One of the most significant factors for the poor prognosis of NSCLC is the treatment resistance for multiple small molecules, including chemotherapeutic and targeted drugs (2, 3).

As an oncogene, epidermal growth factor receptor (EGFR) is frequently mutated and abnormally activated among non-smoking NSCLC patients, driving the progression of NSCLC (4, 5). Hence, the EGFR tyrosine kinase inhibitors (EGFR-TKIs) have been investigated. The representative drug is gefitinib (GE), which is currently used as the first-line targeted therapy drug for NSCLC patients carrying EGFR mutations (6). The initial high efficacy of GE could be observed in specific NSCLC patients. Regrettably, nearly three-quarters of GE-sensitive NSCLC patients ultimately evolve inevitable treatment resistance in a year, resulting in high mortality rates (7). Therefore, overcoming the resistance of GE in NSCLC is becoming a crucial issue and a significant project in NSCLC management.

A few studies have identified the potential mechanisms for GE primary or acquired resistance in NSCLC, including KRAS mutations as well as mutations on EGFR kinase activity sites, ultimately inducing gain of function of such an oncogene, in which the transformation of T790M and C797S in EGFR are the top two forms (8). Moreover, the downstream and bypass signaling aberrant activation, including BRAF fusion and PIK3CA mutation, and cell phenotype transformation, including epithelial–mesenchymal transition (EMT), could also contribute significantly to the GE resistance in NSCLC (9, 10). More recently, the ATP-binding cassette (ABC) transporters family have been discovered to mediate GE treatment sensitivity, in which the breast cancer resistance protein (BCRP) and multidrug resistance protein-7 (MRP-7) play a significant role in GE resistance (11–13). The mechanisms of action on BCRP- and MRP-7-induced GE resistance involve the intrinsic characteristic of the transporter excretion, which relies on the energy of ATP hydrolysis (14, 15). Hence, the discovery of molecules that could inhibit the function or decrease the expression of BCRP and MRP-7 is a potential approach to overcome GE resistance in NSCLC (13, 16).

Meclofenamic acid (MA) is a non-steroidal anti-inflammatory drug (NSAID) that has been reported to promote platinum-mediated kidney injury (17). In our tentative exploration, we found that MA and GE in combination showed synergistic effects on NSCLC cells. Hence, in the present study, we aimed to study the novel effect of MA on GE resistance induced by BCRP and MRP-7. Our findings may provide unexplored therapeutic potentials for the solution of GE drug resistance in NSCLC cells.

## Materials and Methods

### Cell Lines and Cell Culture

The GE-sensitive PC9 cell line and H292 cell line were purchased from the Cellular Institute (Cell Bank) of the Chinese Academy of Science (Shanghai, China). The GE-resistant cells PC9-GR and H292-GR were established by exposing them to increasing concentrations of GE. When the cells could not sustain growth similarly to the parental cell lines under GE treatment, the GE was discarded before the cells restored the growth rate. After over 12 months of GE exposure, the PC9-GR and H292-GR cell lines were successfully generated and maintained in 2 μM of GE. MTT assays were conducted to confirm the resistance characteristic of PC9-GR and H292-GR cells. All the above cell lines were cultured in RPMI 1640 medium containing 10% fetal bovine serum (FBS) incubated in a humid atmosphere containing 5% CO_2_. The resistant sublines were cultured in a GE-free complete medium for at least 2 weeks to eliminate the interference of GE before all the experiments.

### Cell Proliferation Evaluation

Cell proliferation detections were conducted by MTT assays. The PC9, PC9-GR, H292, and H292-GR cells (5×10^3^) were seeded into 96-well plates overnight. Then, different concentrations of GE and MA were treated alone or in combination for 48 h. After incubation, MTT (final concentration: 5 mg/ml) was added to the 96-well plates and incubated at 37°C in the dark for 4 h. The supernatants were discarded, and the formazan was dissolved by DMSO and measured by an iMark microplate reader (Bio-Rad, Hercules, CA, United States). The viabilities were calculated by a percentage relative to the DMSO-treated control group. A GraphPad 8.01 software calculated the median inhibitory concentration (IC_50_) values. The synergistic effects of GE and MA in different NSCLC cells were evaluated by the Chou–Talalay method described previously (18).

### Apoptosis Analysis

Annexin V-FITC and PI synchronous staining were conducted to evaluate apoptotic cells after GE and MA co-administration in GE-resistant NSCLC cell lines. In brief, cells at a density of 5×10^5^ were seeded into 6-well plates; after incubation overnight, different concentrations of GE and MA were co-administrated for 48 h at 37°C. Subsequently, the cells were collected and stained with Annexin V-FITC and PI (5% respectively) for 30 min at RT in the dark. Then, the cells were centrifuged and resuspended in 250 μl of detection buffer and analyzed by MACS Verse flow cytometer (BD Biosciences, San Jose, CA, United States). The qualification of apoptotic cells was analyzed by FlowJo VX software (Tristar, CA, United States).

### GE Accumulation Study

The accumulation of GE in resistant NSCLC cells was evaluated by tritium-labeled GE [GE-T (G), 10 Ci/mmol], purchased from Energy Chemical (Shanghai, China). The cells were plated into 6-well plates overnight and co-administrated with GE-T (G) and MA for 2 h, 24 h, and 48 h. Then, the cells were collected and the liquid scintillation was detected by a Quantulus™ GCT spectrometer (Perkin-Elmer, Waltham, MA, United States). The accumulation rates of GE were calculated *via* a custom standard curve.

### Western Blot

The expression level of EGFR-related downstream pathway molecules, the BCRP and MRP-7 expression level, and FTO and its downstream molecules were evaluated by Western blot assay. Specifically, cells were plated into a 6-well plate overnight and co-administrated with GE and MA. The Triton-X 100 lysis buffer was used to extract the total protein. The concentrations of proteins were qualified by BCA protein Assay Kit (Pierce™, Thermo Fisher Scientific, Waltham, MA, United States). Equal amounts of proteins (20–60 μg) were subjected to 8% to 12% sodium dodecyl sulfate-polyacrylamide gel electrophoresis (SDS-PAGE) before being transferred onto polyvinylidene fluoride (PVDF) membranes (Millipore, Billerica, MA, United States). Non-fat milk (5%) was used as a blocking agent to block the non-specific binding sites of PVDF membranes. The primary antibodies of total EGFR, p-EGFR, p-Akt, p-ERK1/2, BCRP, MRP-7, FTO, and c-Myc were incubated as recommended concentrations with the membrane at 4°C for overnight. Then, the membranes were washed with TBST buffer and incubated with HRP-conjugated secondary antibodies at RT for 1 h. After washing with TBST buffer, the ECL was added to the membrane and an Invitrogen iBright (Thermo Fisher Scientific, Waltham, MA, United States) was used to visualized the protein bands. The relative intensity was calculated by catching the gray level using ImageJ software.

### Quantitative Real-Time PCR

The mRNA expression levels of BCRP, MRP7, FTO, and MYC were determined by qPCR. In brief, the total RNA was extracted from the cells in the presence or absence of MA using TRIzol reagent (Invitrogen, Carlsbad, CA, United States) according to the manufacturer’s instructions. The concentration of total RNA was measured by ultraviolet spectrophotometry. According to the manufacturer’s instructions, complementary DNAs (cDNAs) were transcribed using RevertAid RT (Thermo Fisher Scientific, Waltham, MA, United States). PCR was performed on Real-Time PCR Detection System (Bio-Rad, Hercules, CA, USA) with SYBR™ Green PCR from Thermo Fisher Scientific (Waltham, MA, United States). The 2^–ΔΔCt^ methods calculated the copy number, and samples were run in triplicate and normalized to GAPDH. Then, the expression levels of mRNAs were reported as fold changes versus control. The primers were as follows: For BCRP, the forward primer (5’-3’) was GCTACACCACCTCCTTCTGT, and the reverse primer (5’-3’) was GGAAGAAGAGAACCCCAGCT. For MRP7, the forward primer (5’-3’) was AGAGTACACCTGTGACCTGC, and the reverse primer (5’-3’) was GAGCACCAACAACAGGGAAG. For GAPDH, the forward primer (5’-3’) was GCACCGTCAAGGCTGAGAAC, and the reverse primer (5’-3’) was TGGTGAAGACGCCAGTGGA.

### Tritium-Labeled GE Accumulation Analysis

The parental and drug-resistant cell lines were seeded into 6-well plates overnight. The cells were incubated with tritium-labeled GE (GE-T) in the presence or absence of MA for 2 h, 24 h, 48 h, or 72 h, respectively, at 37°C containing 5% CO_2_. Then, the cells were lysed by Triton X-100 lysis buffer (Beyotime, Shanghai, China). The radioactivity of the total lysis product was measured by a Tri-Card 4910 liquid scintillation analyzer (PerkinElmer, Waltham, MA, United States).

### Cell Membrane Protein Extraction

A membrane and cytoplasm protein isolation kit isolated the cell membrane protein from Beyotime (Shanghai, China). In brief, 5×10^7^ cells were seeded into a 6-cm^2^ dish, then were incubated in the presence or absence of GE and MA for 24 h. The cells were collected after treating with EDTA buffer, centrifuged, and homogenized. The nucleus was discarded by centrifuging at 700*g* for 10 min at 4°C, and the supernatants were collected. The cell membrane was collected by centrifuging at 14,000*g* for 30 min at 4°C. Then, the manufactured lysis buffer was used to extract membrane proteins. The composed membrane proteins were subjected to SDS-PAGE subsequently as aforementioned.

### Dot Blot Assays for m^6^A Level Detection

The dot blot assays for detecting m^6^A levels were conducted as previously described (19). In brief, according to the manufacturer’s instructions, the total RNAs were isolated from the cells in the presence or absence of GE and MA using TRIzol reagent (Invitrogen, Carlsbad, CA, United States). PolyATtract^®^ mRNA Isolation Systems (Promega, Madison, WI, United States) was then used to extract mRNA. The ultraviolet spectrophotometry method was used to determine the concentration of mRNA. Subsequently, the mRNAs were denatured for 3 min at 95°C, followed by cooling in ice directly. The mRNAs were UV cross-linked after being spotted on an Amersham Biosciences Hybond-N+ membrane optimized for nucleic acid transfer (GE Healthcare, Little Chalfont, United Kingdom). The membranes were shed with TBST buffer, followed by blocking with 5% non-fat milk, and hybridized with anti-m6A antibody (Abcam, Cambridge, MA, United States) overnight at 4°C. Then, the membranes were washed with TBST buffer and incubated with HRP-conjugated secondary antibodies at RT for 1 h. After washing with TBST buffer, the ECL was incubated and an Invitrogen iBright (Thermo Fisher Scientific, Waltham, MA, United States). The relative intensity was calculated by catching the gray level using ImageJ software.

### Me-RIP

The methylated RNA immunoprecipitation (Me-RIP) was conducted to detect the m^6^A modification of MYC as previously described (20). In brief, total RNAs were isolated, then the mRNA was further isolated and purified using the TIANSeq mRNA Capture Kit (TIANGEN Biotech, Beijing, China). The anti-M^6^A or anti-IgG (1:100, Abcam, Cambridge, UK) were added and incubated with protein A/G agarose beads (Santa Cruz Biotechnology, Santa Cruz, CA, United States) in IP buffer overnight at 4°C. The eluent buffer was conducted to elute RNA. The phenol-chloroform was used to purify the RNAs. The qPCR was performed to determine the mRNA level of m^6^A MYC.

### Statistical Analysis

Data are expressed as mean ± SD in at least three independent experiments. The significance was determined using one-way ANOVA. GraphPad 8.00 software was used to determine the statistical analysis. *p* < 0.05 was considered statistically significant.

## Results

### The Synergistic Anti-Viability Effects of MA and GE on GE-Resistant Sublines

**Figure 1:**
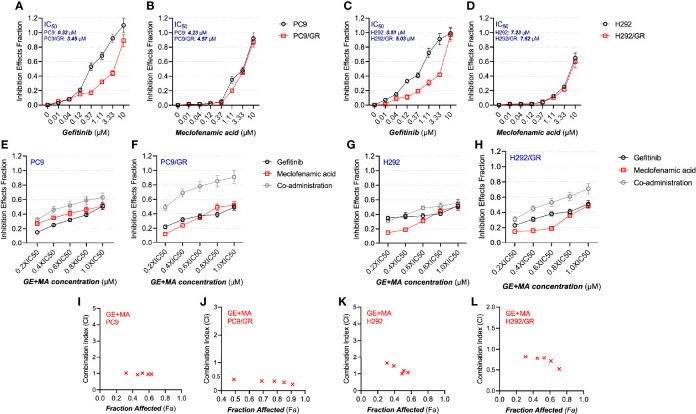
The antiproliferative effects of MA and/or GE on gefitinib-resistant NSCLC cells. **(A–D)** The parental PC9 and H292 as well as gefitinib-resistant PC9/GR and H292/GR cells were treated with various concentrations of GE or MA; the antiproliferative effects were determined by MTT assay following 48 h of incubation. Cells were treated with GE and MA as a single agent or in combination for 48 h, and MTT assays were used to analyze the cell viability. The Chou–Talalay method was conducted to calculate the combination index (CI); a CalcuSyn software were used to analyze the data. **(E–H)** illustrate the combination ratio/viability, while **(I–L)** show the fraction affected/combination index. Data are presented for at least three independent repetitions and were illustrated as mean ± SD.

The potential synergistic anti-proliferation effects of MA and GE were determined by MTT assays and fitted using Chou–Talalay methods as previously described (18). We first detected the antiproliferation characters of MA and GE on parental and resistant NSCLC cells. As illustrated in **Figures 1A–D**, the IC_50_ values of GE on PC9 and PC9/GR cells were 0.32 and 3.45 μM, respectively. In H292 and H292/GR cells, the IC_50_ of GE was 0.51 and 5.03 μM, indicating the confirmed resistance profile of PC9/GR and H292/GR cells. The IC_50_ of MA, in all cell lines, was 4–8 μM.

Next, the drug combination of GE and MA was conducted, and the combination index (CI) was calculated. As shown in **Figures 1E–H**, the different IC_50_ value fractions were undertaken in the combined administration of GE and MA. The CI value calculation results indicated that GE and MA showed potent synergistic effects in GE-resistant cells (PC9/GR and H292/GR). In contrast, no significant synergistic effects of GE and MA were observed in parental cells (**Figures 1I** **–L**). Based on the results of the drug combination, we chose related concentrations for the following experiments: for PC9/GR cells, 2.5 μM GE and 4 μM MA were adopted; for H292/GR cells, 4 μM GE and 6 μM MA were conducted.

### The Combination of GE and MA Significantly Induces Resistant NSCLC Cell Apoptosis

**Figure 2:**
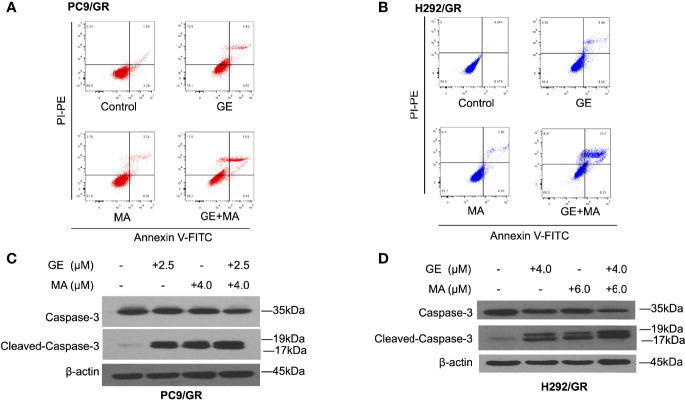
MA and GE synergistically induced caspase-3-associated apoptosis in gefitinib-resistant NSCLC cells. Apoptotic cells in PC9/GR **(A)** and H292/GR **(B)** exposed to the single drugs or combination are presented in dot blot. Western blot results show dynamic caspase-3 face after PC9/GR **(C)** or H292/GR **(D)** exposed to single or combination drugs.

Based on the combination of GE and MA results, different concentrations of GE and MA were co-administrated in PC9/GR and H292/GR cells to evaluate the apoptotic cell population by flow cytometry. As illustrated in **Figures 2A, B**, though single use of GE or MA in PC9/GR and H292/GR cells induced poor apoptosis, the co-administration significantly induced cell apoptosis potently, indicating the potential combined effects of GE and MA. Furthermore, Western blot results showed that the apoptotic effects of GE combination of MA were caspase-3-dependent (**Figures 2C, D**).

### GE-MA Co-Administration Significantly Inhibited EGFR Downstream Pathways

**Figure 3:**
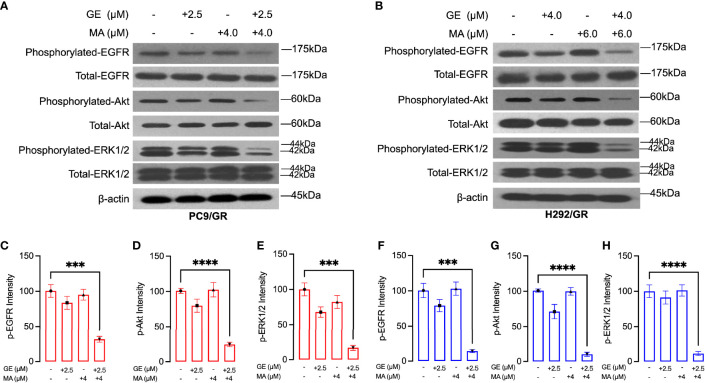
MA and GE synergistically inhibited the activity of the EGFR-related signaling pathway. Western blot results showed the expression of p-EGFR, p-Akt, and p-ERK1/2 in PC9/GR **(A)** and H292/GR **(B)** cells exposed to MA and GE for single or combination use. **(C–H)** showed the statistical analysis of the Western blot results **(A, B)**. Data are presented for at least three independent repetitions and were illustrated as mean ± SD. ***p < 0.001, ****p < 0.0001.

We next evaluated the potential mechanisms of action on the synergistic effects of GE and MA. As shown in **Figure 3A**, in PC9/GR cells, the EGFR-related downstream key molecules were detected by Western blot. GE or MA single use did not significantly induce inactivation of EGFR downstream molecules. However, the combination-treated GE and MA significantly inhibited the phosphorylation of EGFR, Akt, and ERK1/2, which indicated that the synergistic effects of GE and MA might be related to the inactivation of the EGFR molecule pathway. In addition, the co-administration of GE and MA in H292/GR cells also induced a potent decrease of phosphorylated modification of EGFR signaling pathways molecules, which further indicated that the EGFR signaling is related to the synergistic effects of GE and MA on drug-resistant NSCLC cells (**Figure 3**).

### MA Significantly Induced GE Accumulation in GE-Resistant NSCLC Cells

**Figure 4:**
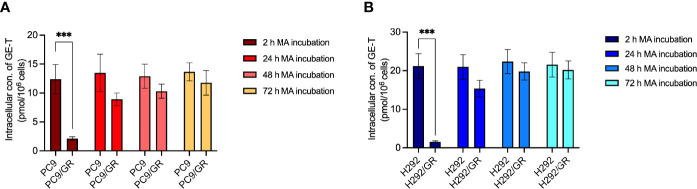
MA significantly enhanced the accumulation of GE in gefitinib-resistant cells. **(A)** The collection of GE in parental PC9 and PC9/GR cells exposed to MA. **(B)** The accumulation of GE in parental H292 and H292/GR cells exposed to MA. Data are presented for at least three independent repetitions and were illustrated as mean ± SD. ***p < 0.001.

Considering the impaired cytotoxicity of GE in resistant NSCLC cells, the synergistic effects may be related to the increased accumulation of GE. Hence, the tritium-labeled GE-based accumulation assays were conducted. As shown in **Figures 4A, B**, short-term incubation of MA (2 h) did not significantly alter GE accumulation in either PC9/GR or H292/GR cells. Interestingly, more prolonged incubation of MA (24 h, 48 h, and 72 h) potently increased the intracellular tritium-labeled GE in PC9/GR and H292/GR cells, but not in their corresponding parental cells.

### MA and the Combination of GE Significantly Decrease the Expression Level of Membrane Drug Efflux Proteins

**Figure 5:**
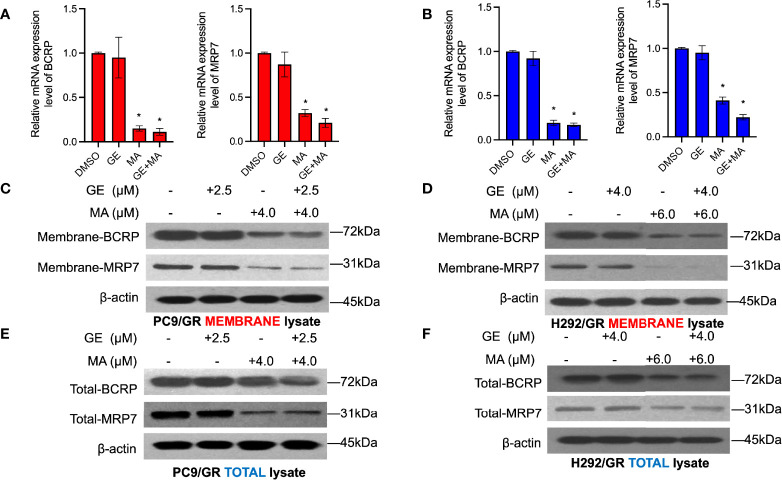
MA and GE synergistically inhibited the expression of BCRP and MRP7. BCRP and MRP7 in PC9/GR **(A)** and H292/GR **(B)** cells. **(C, D)** The membrane protein expression of BCRP and MRP7 in PC9/GR and H292/GR cells after MA and GE for single or in combination exposed. **(E, F)** The total cell lysate protein expression of BCRP and MRP7 in PC9/GR and H292/GR cells after MA and GE for single or in combination exposed. Data are presented for at least three independent repetitions and were illustrated as mean ± SD. *p < 0.05.

We next evaluated whether MA or combination with GE could alter the membrane drug pumps in GE-resistant NSCLC cells. As illustrated in **Figure 5**, the cell membrane of both PC9/GR and H292/GR cells was isolated, and SDS-PAGE detected the expression of MRP7 and BCRP. As illustrated in **Figure 5A**, the expression of MRP7 and BCRP in PC9/GR significantly decreased in MA and MA/GE co-administrated group compared with the control or GE treatment group. In addition, similar results were found in H292/GR cells, where both the BCRP and MRP7 were downregulated by MA or MA/GE co-administrated.

### Co-Administration of MA and GE Downregulated BCRP and MRP7 Expression by Increasing the M6A Modification of MYC

**Figure 6:**
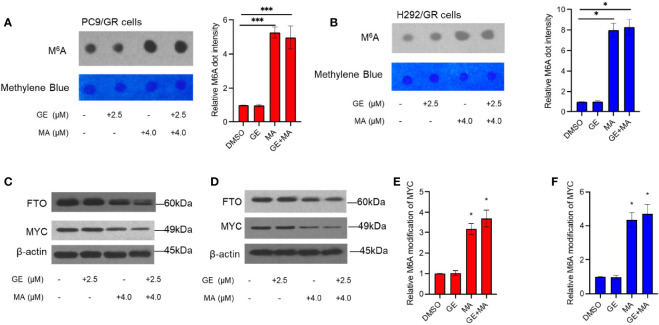
MA and GE in combination upregulated FTO-mediated MYC M6A modification. **(A, B)** Dot blot shows the M6A modification level in PC9/GR and H292/GR cells exposed to MA and GE for a single drug or combination. **(C, D)** Expression of FTO and MYC determined by Western blot assays in PC9/GR and H292/GR cells. **(E, F)** The m6A modification level of MYC cells was assessed by Me-RIP assay in PC9/GR and H292/GR cells exposed to MA and GE for a single drug or in combination. Data are presented for at least three independent repetitions and were illustrated as mean ± SD. *p < 0.05, ***p < 0.001.

The total M6A modification in PC9/GR and H292/GR cells was detected by dot blot assays of M6A. As shown in **Figures 6A, B**, the M6A modification of mRNA in the MA- or MA/GE-treated group significantly increased, indicating that MA or MA/GE co-administration may affect the activity of M6A-related proteins. Moreover, demethylase FTO was significantly downregulated in both PC9/GR and H292/GR cells in MA- and MA/GE-treated groups (**Figures 6C, D**). Furthermore, the transcription factor MYC was significantly decreased in the MA or MA/GE co-treatment group. The Me-RIP results indicated that both MA alone and in combination with the GE group significantly increased the M6A modification of MYC (**Figures 6E, F**).

## Discussion

As a first-line drug for patients with NSCLC, GE has shown great application value in the clinics, but its acquired resistance is a massive obstacle for the further benefits of NSCLC patients (21). The elucidated mechanisms of action on GE-resistant cells are complicated, including EGFR T790M mutation and MET amplification (22). In addition, the increased efflux of GE by BCRP and MRP7 is also responsible for the GE resistance in NSCLC (11, 23). Hence, BCRP and MRP7 are potential targets for reversing GE resistance in NSCLC.

The present study found that MA, an NSAID, showed significant synergistic effects with GE in GE-resistant NSCLC cell lines. Using the Chou–Talalay combination equation, we confirmed that MA and GE showed significantly synergistic effects in GE-selected resistant cells but not in normal NSCLC cells. The caspase-related apoptosis was found after being co-administrated with MA and GE in GE-resistant cell lines. Moreover, the downstream molecules of EGFR pathways were significantly inactivated after combination use, indicating that the administration of MA might enhance the effects of GE. Hence, we further detected whether the accumulation of GE in GE-resistant cell lines increased after MA treatment. The results indicated that MA could significantly enhance the intracellular GE concentration, suggesting that MA may act as a sensitizer of GE in GE-resistant NSCLC cells.

As mentioned above, overexpression of BCRP and MRP7 may be responsible for the resistance of gefitinib in NSCLC cells. Hence, the expression levels of BCRP and MRP7 were detected by Western blot in both cell membrane and total cell lysate. MA and MA/GE coadministration significantly decreased both membrane and whole cell lysate expression of BCRP and MRP7, which explained that MA could increase the accumulation of GE in GE-resistant NSCLC cell lines.

The downregulatory effects of BCRP and MRP7 may be related to the alteration of mRNA; we evaluated the mRNA expression level of both BCRP and MRP7. Our results indicated that MA and MA/GE coadministration significantly decreased the mRNA expression level of BCRP and MRP7. The M6A modification is a hot spot of mRNA modification, which will reduce mRNA stability (24). We hypothesized that the M6A modification might be involved in decreasing BCRP and MRP7 mRNA levels. The results for dot blot assays indicated that the M6A modification level was significantly upregulated. As the main M6A eraser, FTO has been widely accepted to have a significant role in cancers (25, 26). In addition, FTO was also documented to be correlated with the expression of ABC transporters (27). In our Western blot results, we observed that FTO expression significantly decreased after MA or GE/MA co-administration, indicating that the upregulation of global M6A modification may be related to the decreasing of FTO. Myc is a key transcription factor that regulates a series of gene expressions, including BCRP and MRP7 (28, 29). In addition, mRNA of MYC was also documented to be influenced by FTO (30). We used Me-RIP assay to detect the effects of MA or coadministration with GE on M6A modification on MYC mRNA. The results indicated that the M6A modification level was significantly increased after MA or MA/GE co-administration. These results suggest that MA acts as a reversal agent that can enhance resistant NSCLC cells sensitive to the EGFR inhibitor, GE, through FTO/M6A/MYC axis-mediated downregulation of BCRP and MRP7.

ABC transporters have been shown to have a significant role in GE resistance in NSCLC cells. In contrast to active site mutation of EGFR, overexpression of ABC transporters potently decreases the intracellular concentration of substrate drugs, ultimately inducing drug resistance (31). In NSCLC cells, BCRP and MRP7 are two central identified efflux pumps that confer the GE resistance, and GE was also proved to be a substrate of BCRP and MRP7 (13), which makes these two pumps potential targets for overcoming GE resistance in NSCLC. Our findings suggest novel approaches to overcome GE resistance in NSCLC by impeding the expression level of BCRP and MRP7 *via* the FTO/M6A/MYC axis. Nevertheless, further studies need to unfold because the downregulation of efflux pumps could also be influenced by posttranslational modification, including glycosylation and ubiquitination; whether such changes also participate in the synergistic effects of MA and GE remains to be determined.

## Data Availability Statement

The original contributions presented in the study are included in the article/**Supplementary Material**. Further inquiries can be directed to the corresponding author.

## Author Contributions

HC performed the main experiments and analyzed the data. BJ and QZ drafted the manuscript, which HC revised. HC and YZ designed the study and revised the final version of the manuscript. All authors contributed to the article and approved the submitted version.

## Conflict of Interest

The authors declare that the research was conducted in the absence of any commercial or financial relationships that could be construed as a potential conflict of interest.

## Publisher’s Note

All claims expressed in this article are solely those of the authors and do not necessarily represent those of their affiliated organizations, or those of the publisher, the editors and the reviewers. Any product that may be evaluated in this article, or claim that may be made by its manufacturer, is not guaranteed or endorsed by the publisher.
