# Supplementary figures and images for: Meclofenamic Acid Restores Gefinitib Sensitivity by Downregulating Breast Cancer Resistance Protein and Multidrug Resistance Protein 7 via FTO/m6A-Demethylation/c-Myc in Non-Small Cell Lung Cancer
Source: Front Oncol. 2022 Apr 21;12:870636. doi: 10.3389/fonc.2022.870636 (PMC9069108; doi:10.3389/fonc.2022.870636)

## Slide 1
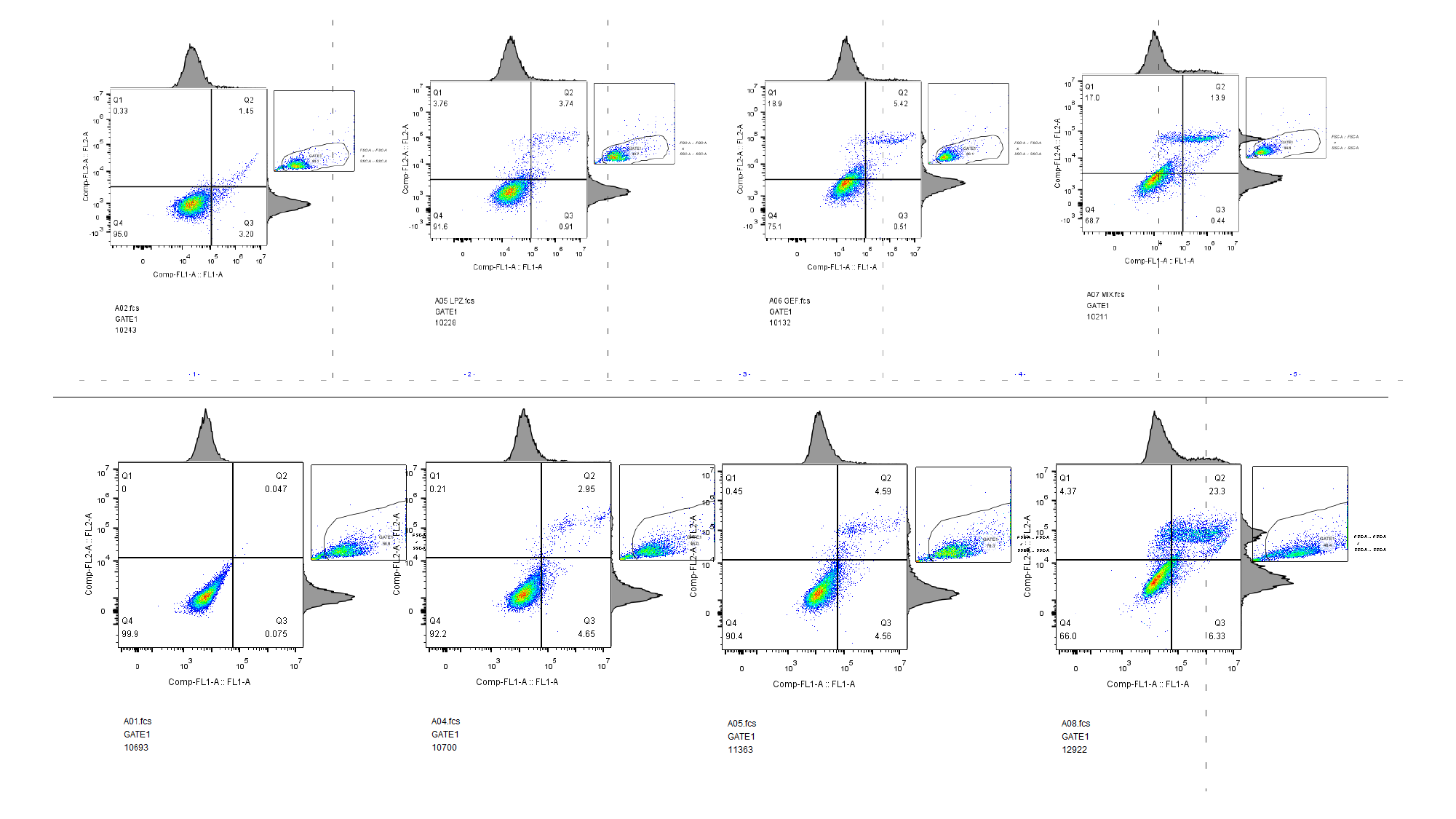

Supplement: Supplementary file 1 [file DataSheet_1.zip › Flowcytometry gating.pptx]

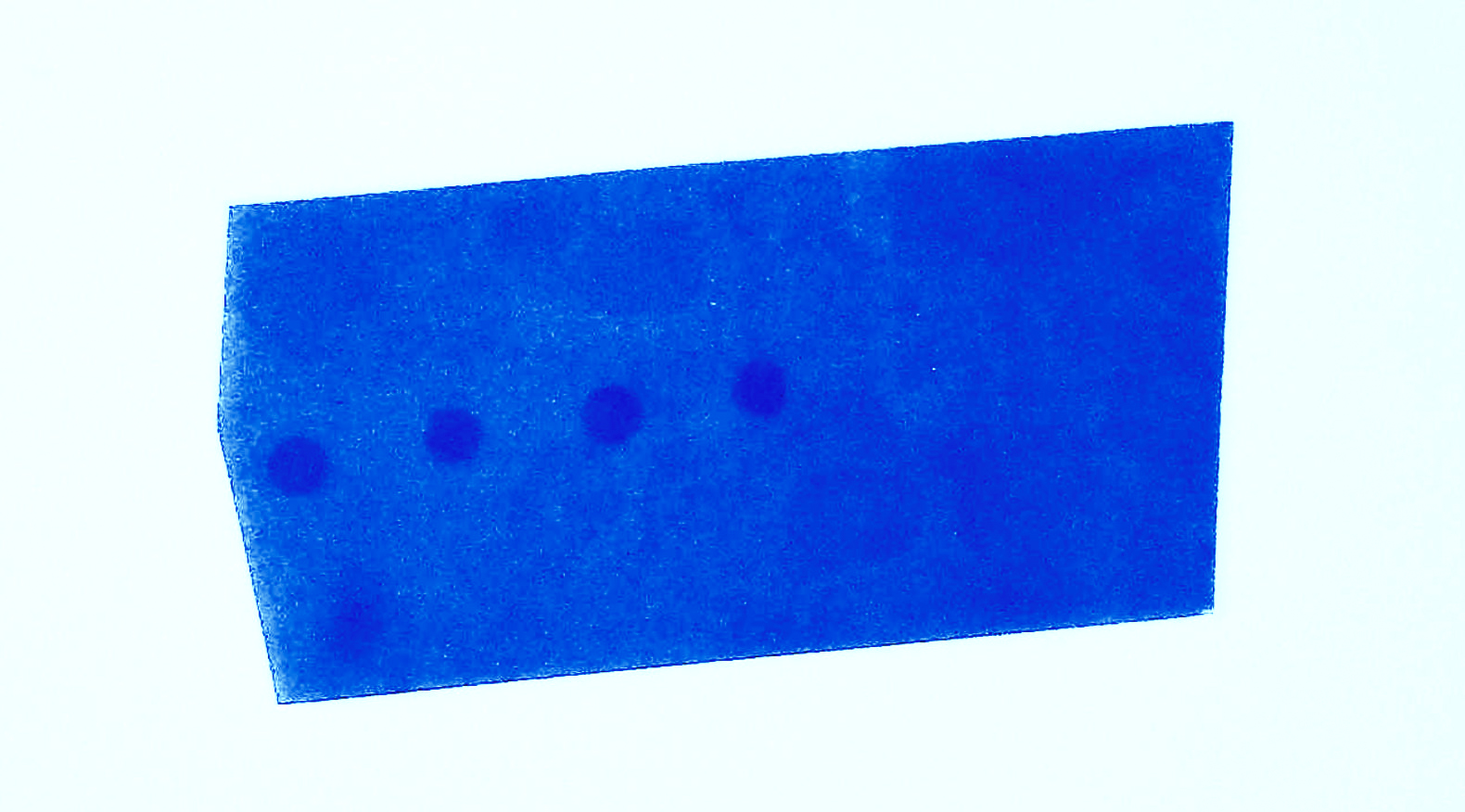

Supplement: Supplementary file 1 [file DataSheet_1.zip › mebluefig6a .jpg]

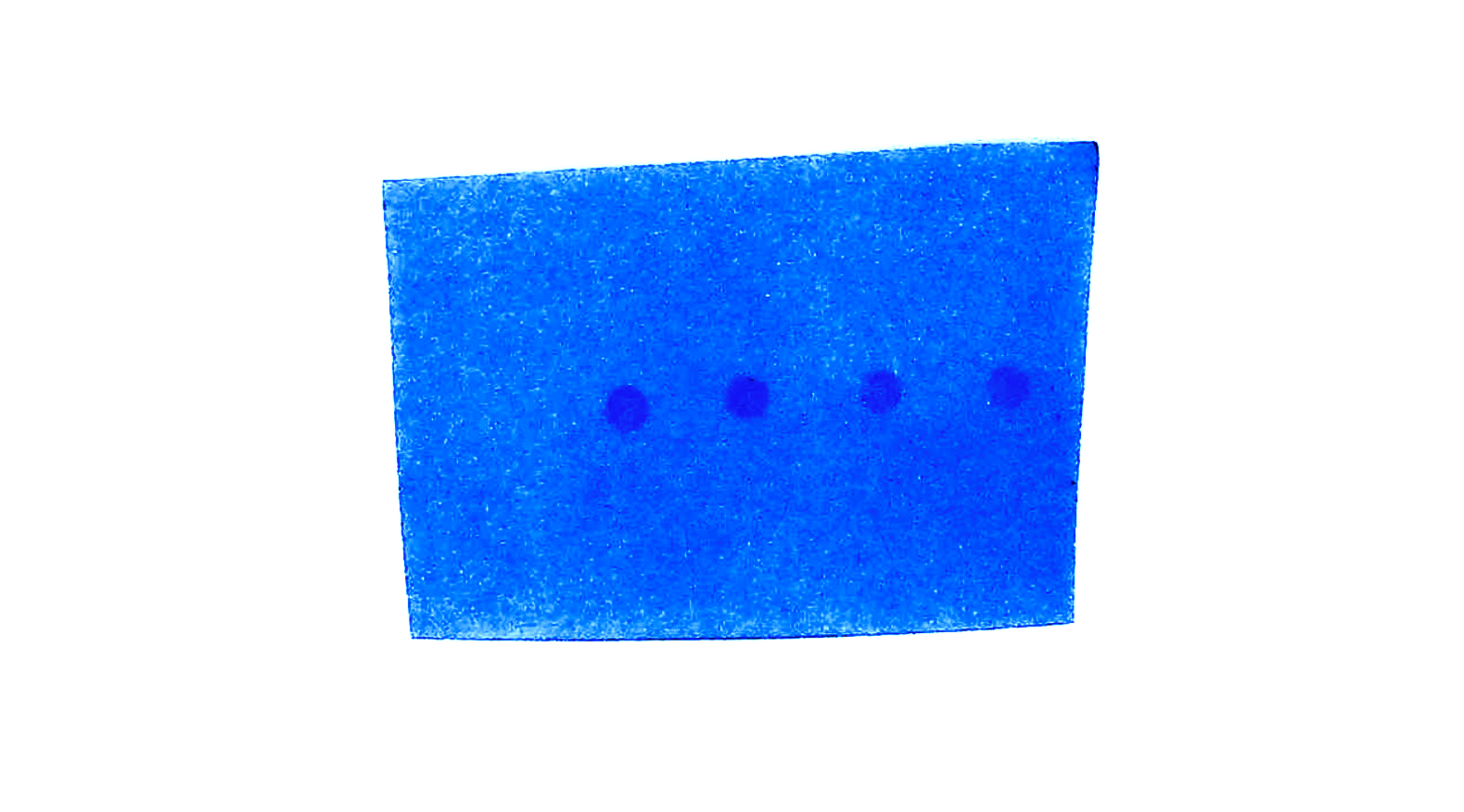

Supplement: Supplementary file 1 [file DataSheet_1.zip › mebluefig6b.jpg]
